# Capturing *Legionella pneumophila* effector enzymes using a ubiquitin derived photo-activatable probe

**DOI:** 10.3389/fmolb.2024.1422034

**Published:** 2024-07-09

**Authors:** Max S. Kloet, Gerbrand J. van der Heden van Noort

**Affiliations:** Department of Cell and Chemical Biology, Leiden University Medical Centre, Leiden, Netherlands

**Keywords:** ADPRibosylation, ubiquitination, covalent probes, post translational modification (PTM), *Legionella*

## Abstract

Upon infection of host cells the *Legionella pneumophila* bacterium releases a multitude of effector enzymes into the host’s cytoplasm that manipulate cellular host pathways, including the host-ubiquitination pathways. The effectors belonging to the SidE-family are involved in non-canonical phosphoribosyl serine ubiquitination (PR-ubiquitination) of host substrate proteins. This results in the recruitment of ER-remodeling proteins and the formation of a Legionella-containing vacuole which is crucial in the onset of legionnaires disease. PR-ubiquitination is a dynamic process reversed by other *Legionella* effectors called Dups. During PR-Ubiquitin phosphodiester hydrolysis Dups form a covalent intermediate with the phosphoribosyl ubiquitylated protein using its active site His67 residue. We envisioned that covalent probes to target *Legionella* effectors could be of value to study these effectors and contribute to deciphering the complex biology of *Legionella* infection. Hence we effectively installed a photo-activatable pyridinium warhead on the 5′-OH of triazole-linked ribosylated ubiquitin allowing crosslinking of the probe to the catalytic histidine residues in *Legionella* SidE or Dup enzymes. *In vitro* tests on recombinantly expressed DupA and SdeA_PDE_ revealed that the probe was able to capture the enzymes covalently upon photo-activation.

## 1 Introduction

Proper cellular homeostasis is tightly regulated by amongst others a wide variety of diverse post-translational modification (PTM) processes. Both ubiquitination and ADPribosylation dictate a highly complex signaling code and intriguingly these two PTMs also regulate each other in processes such as, for example, polyADPribose directed Ubiquitination. In addition ubiquitin (Ub) was found to be ADPribosylated on multiple different amino acid positions and *vice versa* ADPribose has been shown to be ubiquitinated (Yang et al., 2017; Buch-Larsen et al., 2020; Zhu et al., 2022; Zhu et al., 2023). This post-translational interplay has important roles in for instance bacterial infection. The discovery of unconventional serine ubiquitination by *Legionella pneumophila* SidE effector enzymes, in which Ub^ADPr^ is formed as intermediate, has gained much interest (Bhogaraju et al., 2016; Qiu et al., 2016). In this process ADP-ribosylation of Arg42 of Ub serves as a first step, followed by coupling to a serine residue in a target protein while expelling adenosine-mono-phosphate, resulting in a phosphoribosyl link between the host protein and Ub (Bhogaraju et al., 2016; Kotewicz et al., 2017; Akturk et al., 2018; Dong et al., 2018). A multitude of *Legionella* enzymes is involved in regulating phosphoribosyl-ubiquitination as Dup enzymes have been shown to release this phophoribosyl linked Ub cargo from the target protein (Wan et al., 2019; Shin et al., 2020), the *Legionella* MavL effector is a glycohydrolase that cleaves ADPr from Ub (Zhang et al., 2024), LnaB is a phosphoryl-AMPylase that restores Ub^ADPr^ (Wang et al., 2024) and SidJ-effectors regulate SidE activity (Jeong et al., 2015; Bhogaraju et al., 2019; Adams et al., 2021). The bacterium hence tightly regulates this unconventional ubiquitination process to maintain dynamic control over part of the host cells ubiquitinome. (Bio)chemical methodology and tools to investigate ADPribosylation of Ub and its role in cellular biology are emerging. (Puvar et al., 2020; Kim et al., 2021; Voorneveld et al., 2022; Kloet et al., 2024).

Histidine residues occur infrequently in nature (<2.5%), but often play a critical role as key residues in over 15% of enzyme active sites (Bartlett et al., 2002). Histidines are also present in for instance the active sites of the ubiquitin-specific protease- (USP) and histone deacetylase enzyme families as well as in the SidE ligase and Dup hydrolase families in *L. pneumophila* (Haberland et al., 2009; Komander et al., 2009; Kalayil et al., 2018). The phospho-diesterase (PDE) domain of SidE proteins, responsible for ligating the serine of the targeted substrate protein to the ADPribosylated Ub, as well as the Dup hydrolases, use a similar active site containing a Glu-His-His triad for catalysis. Covalent reacting activity-based probes have proven to be very useful in interrogation of the Ub system (de Jong et al., 2012; Ekkebus et al., 2013; Flierman et al., 2016; Mulder et al., 2016). Most of these probes rely on reactivity of the cysteine residues in the active sites of Ub-proteases or -ligases towards Michael acceptor type “warheads”. To effectively design a probe that targets catalytic histidine residues, it is crucial to incorporate an appropriate reactive moiety tailored to the reactivity of histidine residues. The challenging aspect of targeting histidine lies in its moderate nucleophilicity compared to other residues like cysteine or lysine (Takahashi, 1976; Roberts et al., 1977). Diethyl vinylphosphonate and 2-cyclohexenone have been reported to target solvent exposed His residues that, although moderately reactive, do show chemo-selectivity towards histidine (Joshi and Rai, 2019). Vinyl sulfonates were also investigated and proven to be highly reactive towards His yet also showed cross-reactivity to Lys and Cys residues (Li et al., 2019; Dalton and Campos, 2020). Specific targeting of active site histidine residues is even more challenging due to their embedding within the protein structure, often requiring ligand-directed approaches for enzyme targeting (Liu et al., 1998; Tsukiji et al., 2009; De Saint Germain et al., 2016; Yoshizawa et al., 2018; Tamura and Hamachi, 2019).

**FIGURE 1:**
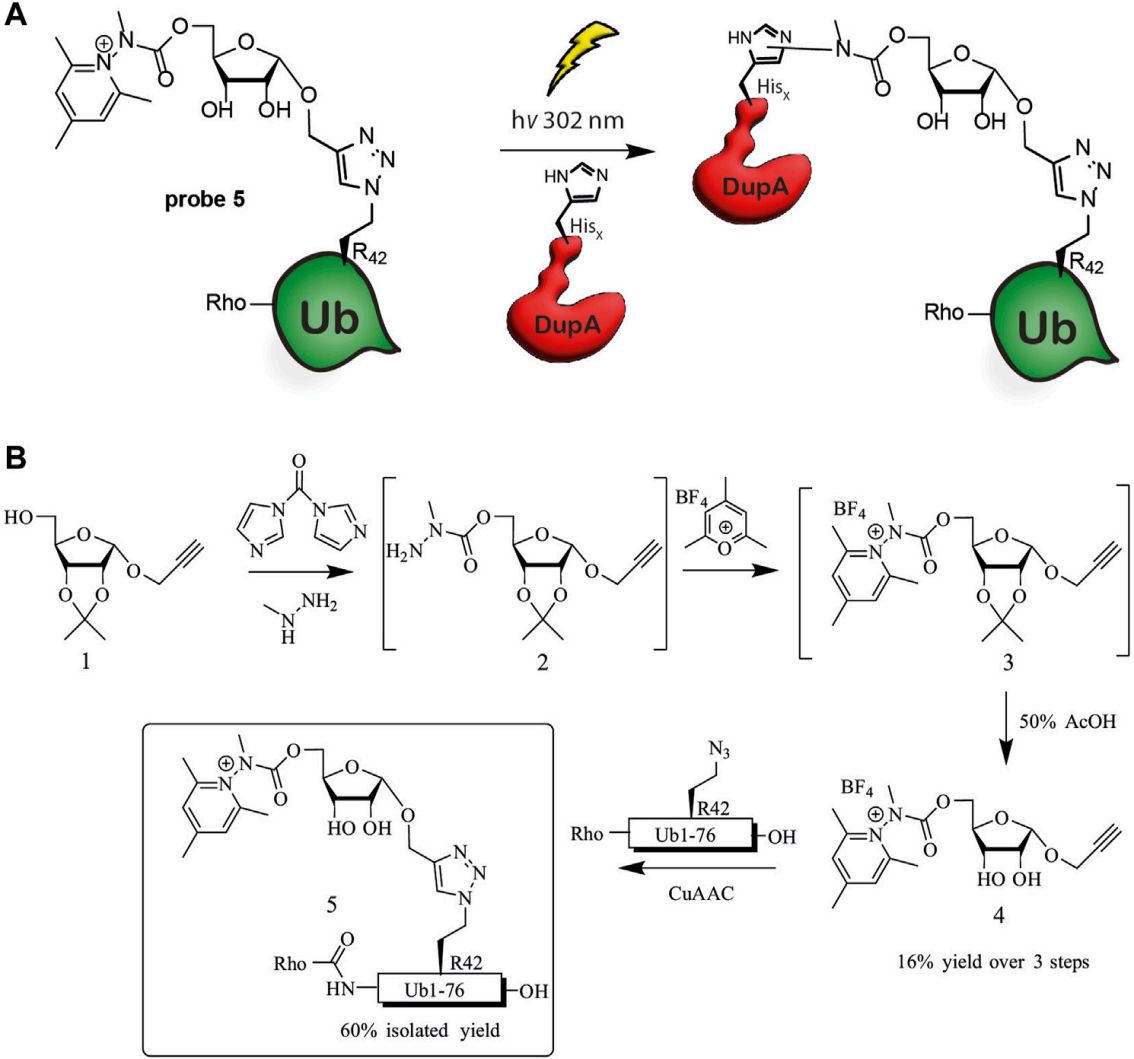
Ub-pyridinium probe **5**. **(A)** Schematic representation of cross-coupling of probe **5** and *Legionella* enzyme DupA, **(B)** Synthesis scheme of probe **5**.

Based on our experience with the highly reactive vinyl sulfonate probes covalently targeting Cys196 of *Legionella* DupA and DupB, we set out to induce a His-selective reaction in the active site of DupA using a similar ligand directed approach (Kloet et al., 2024). Hacker and co-workers observed significant labeling of histidine residues in the proteome when using an Katritzky-type pyridinium warhead (Bapat et al., 1976) upon performing photo-cross-coupling in bacterial lysate (Zanon et al., 2021). The here presented approach entails a ligand directed approach of a phosphoribose-Ub substrate mimic equipped with a pyridinium His-reactive warhead to the active site of *Legionella* effectors based on their affinity for (phosphoribosyl)ubiquitin intended to induce a covalent-bond forming reaction with one of the two active site His residues (His67 and His189) upon photo-activation (Figure 1A).

## 2 Materials and methods

### 2.1 Chemical synthesis of probe 5

A detailed description is provided in the Supplementary Material.

### 2.2 HR-MS measurements

LC-MS measurements were conducted on a Waters ACQUITY UPLC H-class System equipped with a Waters ACQUITY Quaternary Solvent Manager (QSM), Waters ACQUITY UPLC Photodiode Array (PDA) eλ Detector (λ = 210–800 nm), Waters ACQUITY UPLC Protein BEH C18 column (1.7 μM, 2.1 × 50 mm) and a Waters XEVO-G2 XS Q-Time-of-flight mass spectrometer equipped with an electrospray ion source in positive mode (Capillary Voltage: 0.5 kV, desolvation gas flow: 900 L/h, desolvation gas temperature: 500°C, source temperature: 130°C, probe angle: 9.5) with a resolution of R = 22,000 (m/z = 500-2000) in ES + mode. Samples were run for 3 min at 40°C using 2 mobile phases: A: MQ + 0.1% formic acid, B: MeCN + 0.1% formic acid. Gradient: 0%–95% B at a flow rate of 0.5 mL/min. Data processing was performed using Waters MassLynx Mass Spectrometry Software 4.1 (deconvolution with MaxEnt1 function).

### 2.3 Photochemical cross-coupling of pyridinium probe 5 to recombinant enzymes

To a 96-well plate containing buffer (20 mM TRIS, 150 mM NaCl, pH 7.6) the enzyme (DupA, SdeA, NRK-1, UCHL-3 or USP-21, 10 μM), probe **5** or azido-Ub (100 μM, 10 eq.) and optionally an additive (GSH, 1 mM or 300 μM) were added. The reactions were performed in a total volume of 50 μL and preincubated on ice for 15 min before irradiation. Irradiation of the reaction mixture was conducted using a 302 nm UV lamp (Analytikjena, UVP 3UV Lamp, 8 W), positioned on top of the 96-wells plate for the indicated time. The reaction mixture was then analyzed directly via LC/MS or SDS-PAGE.

## 3 Results

### 3.1 Synthesis of photo-activatable Ub pyridinium probe (5)

We envisioned construction of probe **5** through a copper(I) catalyzed azide-alkyne cyclo-addition (CuAAC) between a propargyl modified riboside equipped with the Katrizky warhead and an azide modified synthetic Ub protein (Figure 1B). Synthesis was initiated from previously reported ribose **1**, which is equipped with an 1′-anomeric propargyl group and 2′, 3′-isopropylidene protection (Kloet et al., 2024). Activation of the primary alcohol in **1** by 1,1-carbonyldiimidazole (CDI) and subsequent substitution by methyl hydrazine afforded the crude methyl hydrazide **2** (Greulich et al., 2015; Tower et al., 2020). Treating this compound with 2,4,6-trimethylpyrylium tetrafluoroborate in ethanol effectively formed the pyridinium riboside **3**, which was used as crude in the next step (Katritzky et al., 1980). The subsequent deprotection of the isopropylidene proceeded uneventfully and isolation by preparative-HPLC yielded **4** in 16% over three steps. The Rho-Ub (Arg42 to azido homoalanine) mutant was prepared using solid phase peptide synthesis as described earlier (Kloet et al., 2024). To finalize synthesis of probe **5**, deprotected riboside **4** was conjugated to the Rho-Ub mutant through CuAAC, affording the pyridinium riboside ubiquitin probe **5**. The rhodamine fluorophore was attached to the N-terminus of Ub during solid phase peptide synthesis in order to visualize DupA-probe **5** complex formation using in-gel fluorescence.

### 3.2 Probe photo-activation and analysis of probe 5-DupA cross-coupling by mass spectrometry

To assess photo-activation of probe **5** we irradiated the probe (302 nm) for 1 h and activation of the *N*-*N* bond was observed using high-resolution mass-spectrometry, as indicated by loss of the pyridinium moiety (HR-MS: deconvoluted mass = 9.117 Da, Δ = −120 Da) and concomitant loss of the carbamate moiety (HR-MS: deconvoluted mass = 9.060, Δ = −177), Supplementary Figure S1A). However, noise is observed in the <6.000 Da range, potentially attributed to ROS-induced oxidation and photodegradation of ubiquitin, as similarly observed by Tower et al., 2020 using the pyridinium warhead in a Katritzky-type PET cross-coupling. Increased product formation has been reported when adding glutathione (GSH) to the PET cross-coupling as reactive oxygen species (ROS) scavenger (Forman et al., 2009; Tower et al., 2020). Indeed, the addition of GSH (1 mM) resulted in cleaner activation of the probe, indicating a protective role of the additive. However, a covalent adduct between **5** and GSH was observed as side reaction (HR-MS: deconvoluted mass = 9.423 Da/9.380 Da, Supplementary Figure S1B), a reaction also reported earlier (Fava et al., 1967; Tower et al., 2020). We lowered the GSH concentration to 300 µM and followed activation of the probe after 1, 15, 30, and 60 min (Supplementary Figure S2). Whereas significant amounts of unactivated probe remain up to 30 min irradiation, after 60 min almost all probe has been activated.

After showing activation of the probe to be effective, the next objective was to photo-cross-link Ub probe **5** to recombinant DupA. First, DupA was incubated with probe **5** (10 eq.) and irradiated for 1 h in absence of GSH and moderate complex formation could be detected using mass-spectrometry (Supplementary Figure S3A. HR-MS: deconvoluted mass = 48.561 Da). In the absence of reductive agents multiple presumably oxidized states of the complex but also DupA alone could be observed, complicating analysis. Subsequently, when including GSH (1 mM) as additive in the photo-induced reaction of probe **5** and DupA significantly less conjugate formation and substantial formation of a DupA-GSH adduct (HR-MS: deconvoluted mass = 39.763, Δ = +320) were detected (Supplementary Figure S3B **)**. Although mass spectrometric analysis of the crosslinking reaction is not ideal, we could conclude that photoactivation of the probe is feasible and the formation of the DupA-probe **5** conjugate could be effected. Additionally, GSH as additive has a favorable protective ROS scavenging role, yet also seems to impede the cross-coupling efficiency of Ub probe **5** to DupA.

### 3.3 SDS-PAGE analysis of probe 5-DupA and -SdeA_PDE_ photo-cross-linked conjugates

**FIGURE 2:**
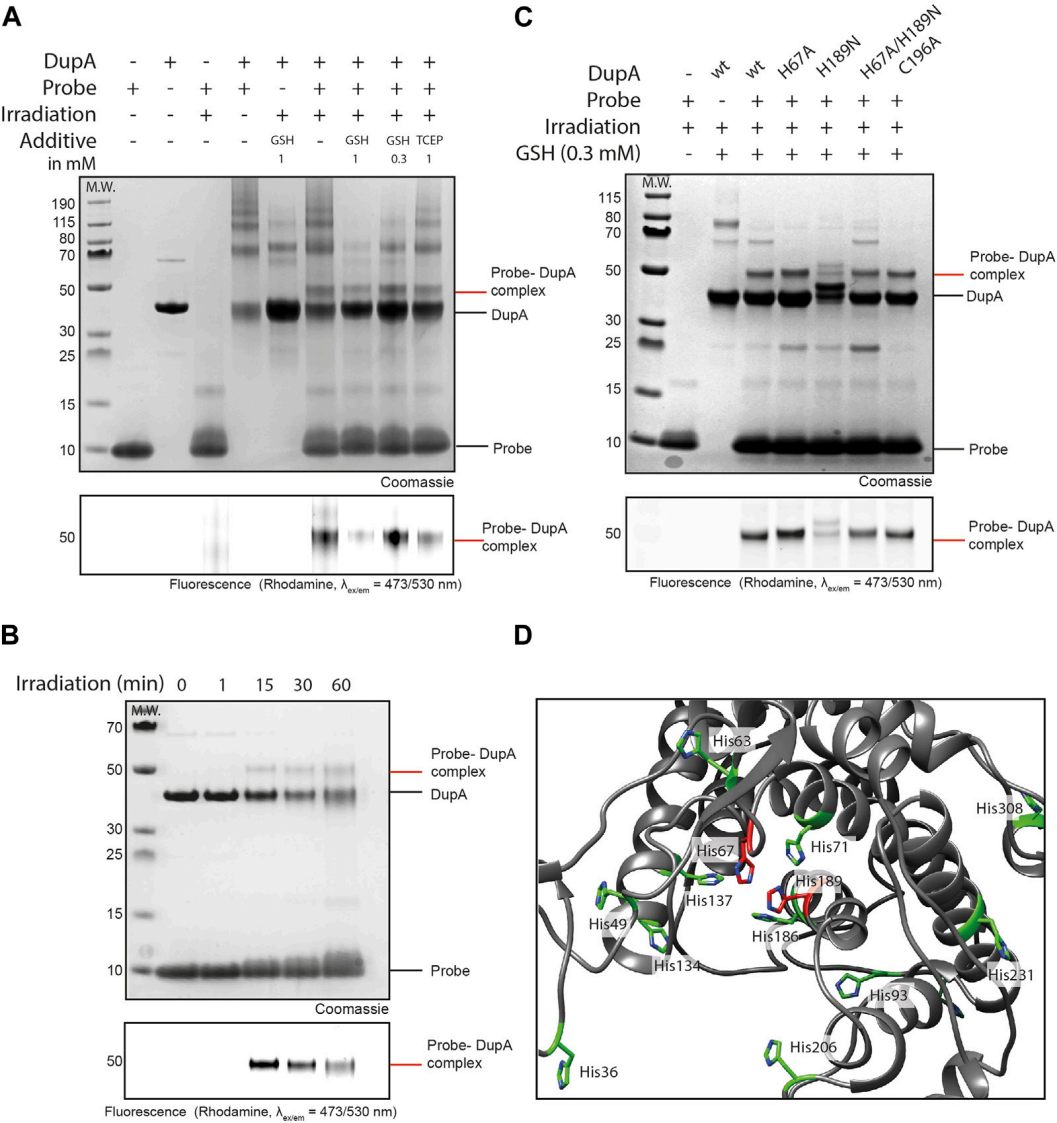
Ub-pyridinium probe **5** crosslinks to DupA upon photo-activation. **(A)** SDS-PAGE gel showing complex formation between probes **5** and DupA wt. 10 equivalents of probe was used and GSH and TCEP were screened as additives. Irradiation for 60 min. Upper panel: Coomassie stained, bottom panel: Rhodamine fluorescence scan (λ_ex/em_ = 473/530 nm). **(B)** SDS-PAGE gel showing complex formation between probe **5** and DupA wt. 10 equivalents of probe in the presence of GSH (300 μM) and irradiation for indicated times. **(C)** Mutagenesis on DupA and reactivity of the mutants to probe **5** in the presence of GSH (300 μM) as additive and irradiation for 15 min. **(D)** Crystal structure of DupA wt (PDB: 6B7O) (grey) highlighting all histidine residues (green) and catalytic histidines (red).

To more efficiently visualize complex formation, a gel-based analysis of the reaction between probe **5** and target protein DupA was conducted (Figure 2A). We screened the above used concentrations of GSH as well as the non-thiol reductant TCEP to investigate the impact on conjugate formation and ROS-protection. Without additive, decent crosslinking was observed, as visualized by Coomassie staining and a rhodamine fluorescence scan. In contrast, irradiating the Rho-Ub (Arg42 to azido homoalanine) mutant that does not contain the photo-activatable group did not result in formation of the crosslinked Ub-DupA complex (Supplementary Figure S4). Consistent with the MS results, 1 mM of GSH proved not to be beneficial for product formation, however seemed to be protective to enzyme integrity as reflected by a reduction of high molecular weight smears. Lowering the GSH concentration (300 μM) improved product formation, albeit with a slight increase in the formation of high molecular weight aggregates compared to the 1 mM GSH conditions. These aggregates are also present when DupA itself is irradiated and hence are not caused by the probe. Addition of tris (2-carboxyethyl)phosphine (TCEP), a reducing agent not acting as a ROS scavenger did not prevent smear formation and hence is no suitable alternative in this reaction. Overall, decreasing the GSH concentration from 1 mM to 300 μM increased the conversion of the photo-cross-coupling while suppressing side product formation. We subsequently followed complex formation over time and although 60 min irradiation leads to a larger conversion into the probe-DupA complex also significant photo-degradation can be seen. Hence the 15 min irradiation time-point was selected as compromise between optimal crosslinking efficiency and preserving protein integrity (Figure 2C).

To investigate whether probe **5** reacts with the active site triad His67 or His189, we monitored complex formation on DupA-(His67 to Ala), DupA–(His189 to Asn) and DupA-(His67 to Ala/His189 to Asn) mutants. Photo-cross-linking of the probe to the DupA (His189 to Asn) mutant demonstrated a reduction in labelling compared to DupA wild type (wt) and all other mutants, as evidenced by SDS-PAGE Coomassie and a fluorescence scan (Figure 2B). This reduction indicates a disability of the probe to react with the DupA (His189 to Asn) mutant, although some residual labeling is still visible for this mutant. The double mutant DupA (His67 to Ala/His189 to Asn) however does not show a marked reduction in labelling efficiency. Interestingly, upon closer examination of the crystal structure (PDB: 6B7O), multiple additional histidine residues in the catalytic cleft were observed (Figure 2D). The presence of other His residues might explain residual labeling in all samples, as upon mutagenesis of the active site residues the probe could still potentially bind one of the other His in the same pocket. Of note, the Cys196 to Ala mutant was labeled to comparable extent as DupA wt, suggesting the probe has no reactivity towards the Cys residue.

**FIGURE 3:**
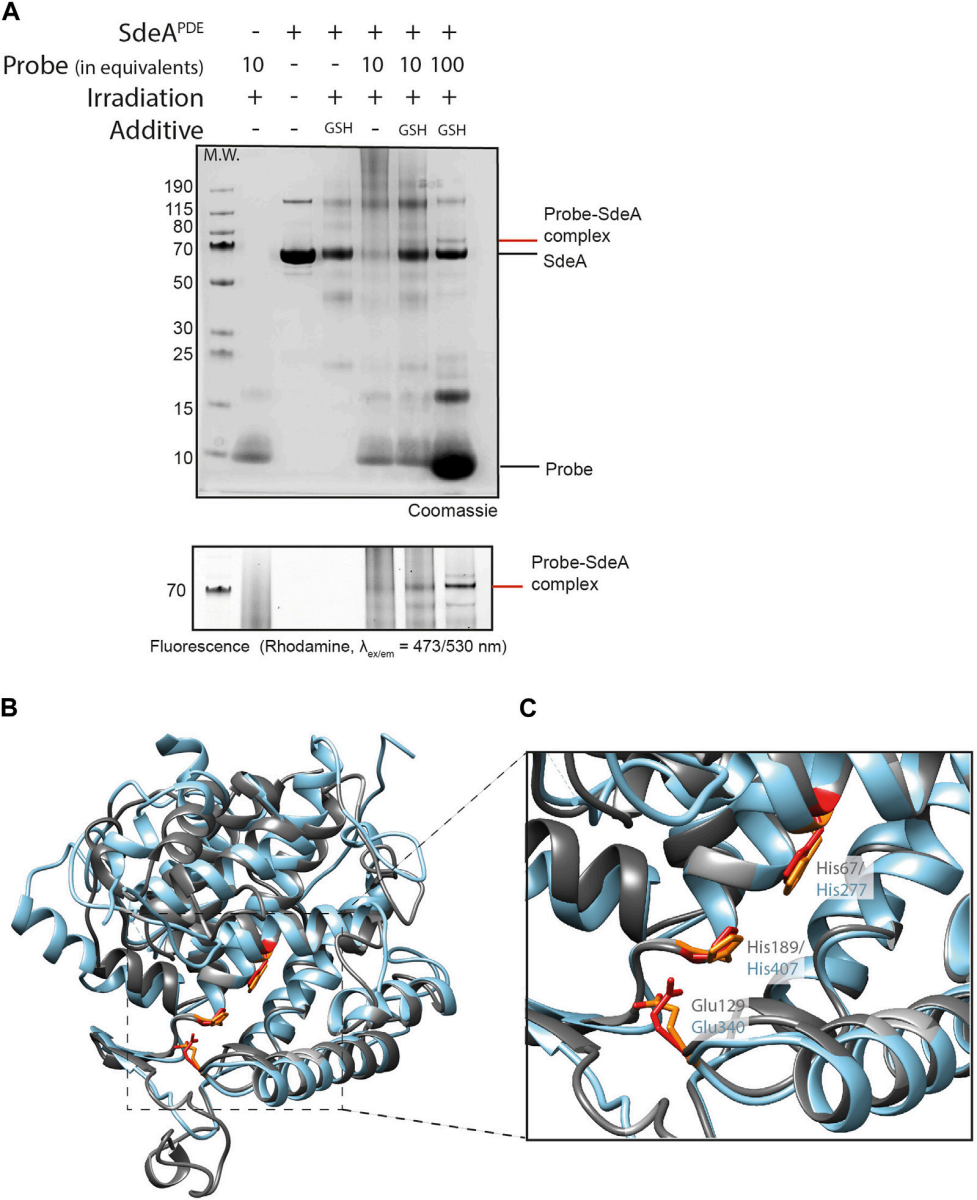
Ub-pyridinium probe **5** binds SdeA PDE upon photo-activation. **(A)** SDS-PAGE showing complex formation between probes **5** and SdeA PDE (10 eq. or 100 eq. of probe). Irradiation was performed for 1 h at 302 nm. Upper panel: Coomassie stained, bottom panel, zoom in on Rhodamine fluorescence scan (_ex/em_ = 473/530 nm). **(B)** Superimposition of DupA (PDB:6B7O) (grey) and the PDE domain of SdeA (PDB:6G0C) (light blue), **(C)** Zoom in on the catalytic triads of DupA and SdeA_PDE_.

DupA and SdeA_PDE_ are structurally homologous featuring a similar catalytic triad (Figures 3B,C), and consequently we set out to verify if an active site His in the PDE domain of SdeA could also be targeted using probe **5**. Initially, only small traces of probe **5**-SdeA_PDE_ conjugate formation were observed using 10 equivalents of probe. Gratifyingly, increasing the amount of probe to 100 equivalents improved the conversion and resulted in a clear visual band above the 70 kDa marker, the expected molecular weight region of the conjugate (Figure 3A). Moreover, this band is clearly detectable in the fluorescence channel and not observed in the controls.

To provide an indication of selectivity of our probe to *Legionella* effector enzymes, the pyridinium probe was reacted with enzymes NRK-1, UCHL-3 and USP-21. When incubating **5** with NRK-1, which could potentially cross-react due to recognition of the riboside-moiety in the probe as a nucleoside-like substrate, no conjugate formation was observed after irradiation (Supplementary Figure S5). Subsequently, probe **5** was irradiated in the presence of deubiquitinating proteases, UCHL3 or USP21. As these DUBs recognize ubiquitin as substrate and contain a histidine in their active site, they may react as off-targets. Interestingly, UCHL-3 exhibited some reactivity to the probe, whereas USP-21 showed no reactivity (Supplementary Figure S5). To verify the extent of off-target reactivity and validate crosslinking potential of probe **5** towards DupA in a more complex environment, we incubated the probe in HEK cell lysate containing spiked in DupA and performed photo-activation followed by SDS-PAGE analysis (Supplementary Figure S6). The DupA-probe conjugate can be appreciated in the fluorescent channel running at the same apparent molecular weight as the complex formed in buffer, whereas either omission of spiking in DupA or irradiation does not lead to the complex being formed in the lysate.

## 4 Discussion

We here show a methodology to incorporate a Katritzky-type pyridinium warhead in a Ub-based probe to induce a photo-cross-coupling that could potentially be used to selectively label His residues in the active site of *Legionella* effector enzymes involved in the unconventional phosphoribosyl ubiquitination pathway. Installing this warhead on the primary alcohol of an anomeric alkyne equipped riboside proved to be effective in 16% over 3 steps after HPLC-purification. Subsequently, conjugation through CuAAC of the formed pyridinium riboside **4** to a Rhodamine labelled Ub_76_ (Arg42 to azido homoalanine) mutant resulted in the Ub-phosphoribose mimicking probe **5**. Photoactivation of the probe was initially analyzed by HR-MS, showing near-complete activation of the probe after 1 h of irradiation (302 nm). However, due to multiple oxidations and potential photo-degradation occurring during the reaction, analysis of the complex formation between probe **5** and DupA wt proved to be difficult using mass spectrometry. Gratifyingly, in-gel fluorescence and Coomassie staining on SDS-PAGE effectively visualized the formation of the probe-DupA conjugate after irradiation. To our knowledge this is the first example of the pyridinium based photochemical reaction to cross-couple two proteins. Optimization of the reaction conditions involved the application of GSH as additive, indicating a protective role of GSH, consistent with findings by Tower et al. (2020). In order to pinpoint which amino acid in DupA reacts with probe **5**, the photoreaction was performed on DupA active site mutants (His67 to Ala), (His189 to Asn) and (His67 to Ala/His189 to Asn). Strikingly, compared to the DupA wt, reduced conversion is observed for the His189 to Asn mutant, whereas all other mutants including the Cys196 to Ala mutant retained the same level of reactivity towards the probe. Taken together, this initial data is inconclusive to determine the binding site of probe **5**. Although reactivity of the probe towards the single His189 mutant is reduced, the double His67/His189 mutant labels comparably to wildtype DupA. Due to the presence of other His residues not taken along in this mutational studies, it cannot be excluded that upon mutating one specific His residue the probe is redirected towards another His residue. The cross-coupling of the probe to SdeA_PDE_ proved to be effective as well, although the observed conversion is modest or requires a larger excess of probe. Although both SidE and Dup *Legionella* effectors thus react with probe **5**, the probe also labelled recombinant UCHL-3, suggesting potential off-target reactivity. Performing crosslinking in HEK cell lysate by adding DupA and probe highlights the translatability of this procedure to more complex biological systems. In conclusion the probe demonstrated effective photo-cross-coupling to the *Legionella* effectors DupA and SdeA_PDE_ and holds promise to monitor *Legionella* effector enzymes involved in PR-ubiquitination in *Legionella* infected cells.

## Data Availability

The raw data supporting the conclusions of this article will be made available by the authors, without undue reservation.
