# Supplementary material for: Capturing Legionella pneumophila effector enzymes using a ubiquitin derived photo-activatable probe
Source: Front Mol Biosci. 2024 Jul 9;11:1422034. doi: 10.3389/fmolb.2024.1422034 (PMC11263097; doi:10.3389/fmolb.2024.1422034)
Supplement: Supplementary file 1 [file DataSheet1.PDF]

Capturing Legionella Effector Enzymes Using a Ubiquitin Derived Photo-Activatable Probe

Max S. Kloet and Gerbrand J. van der Heden van Noort*

Department of Cell and Chemical Biology, Leiden University Medical Centre, Leiden, The Netherlands

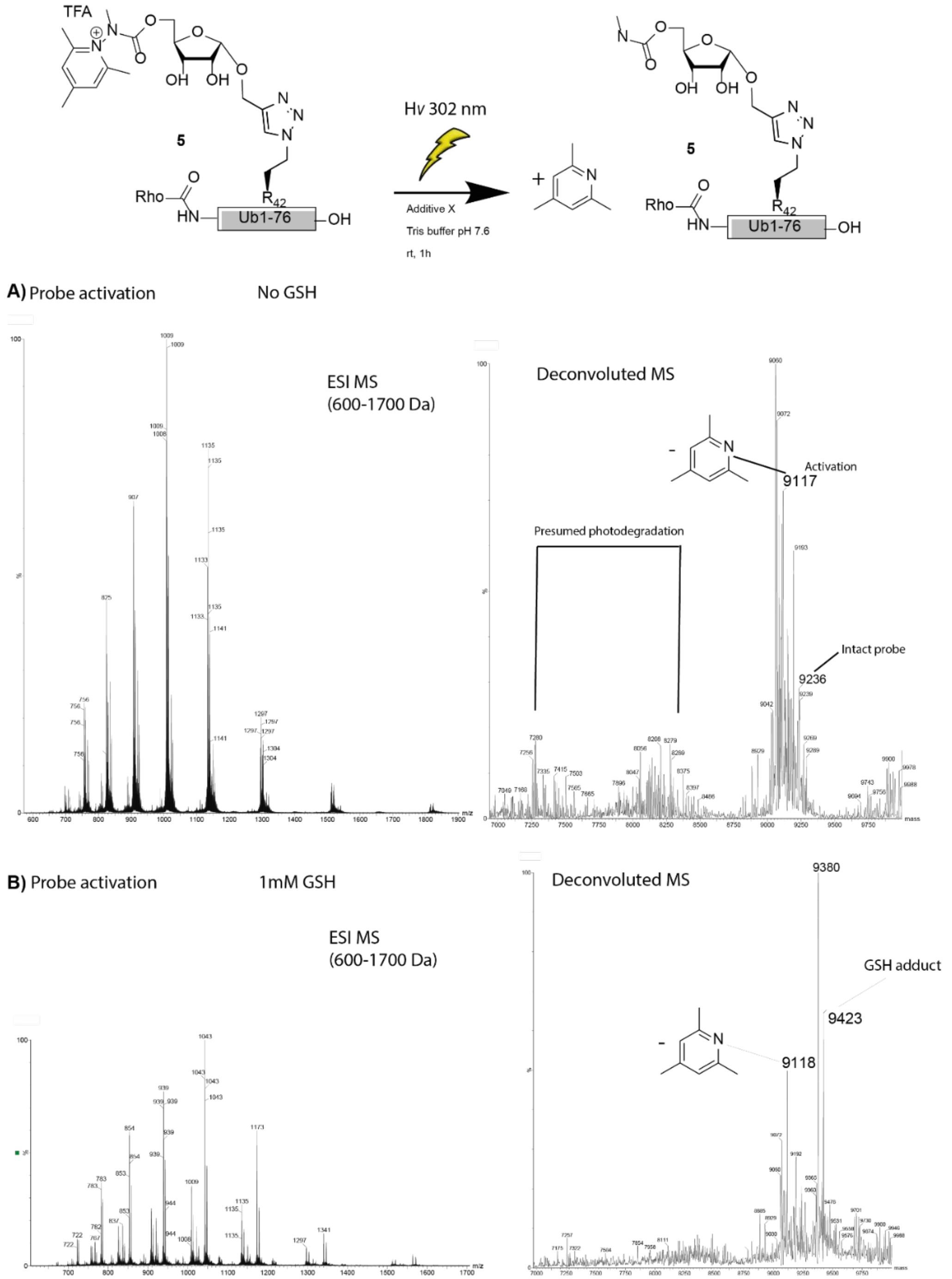

Supplementary Fig. 1. HRMS analysis of the activation of probe **5**. **A**) HRMS of the probe activation without GSH as additive. Probe activation is observed via loss of the pyridinium moiety (M + H^+ = 9117, Δ = 120). Some residual starting material (M + H^+ = 9236) and presumed photodegradation products are as well detected, **B**) photoactivation of **5** in presence of GSH (1 mM). Probe activation (M + H^+ = 9117, Δ = 120) as well as GSH addition (M + H^+ = 9423, Δ = 185, concomitant loss of pyridinium moiety) are observed. The irradiation is performed for 1 hour at 302 nm.

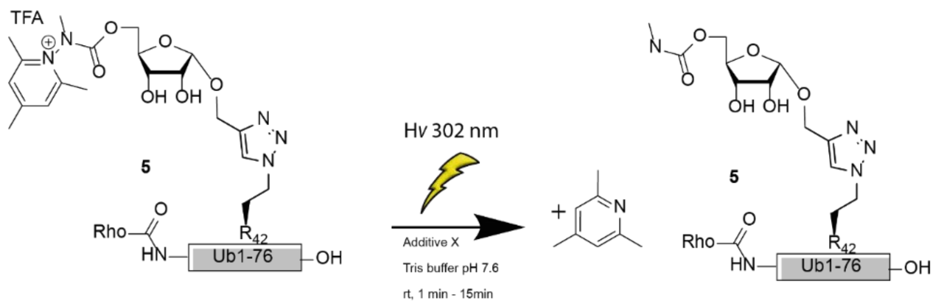

A) 1 min 300 µM GSH

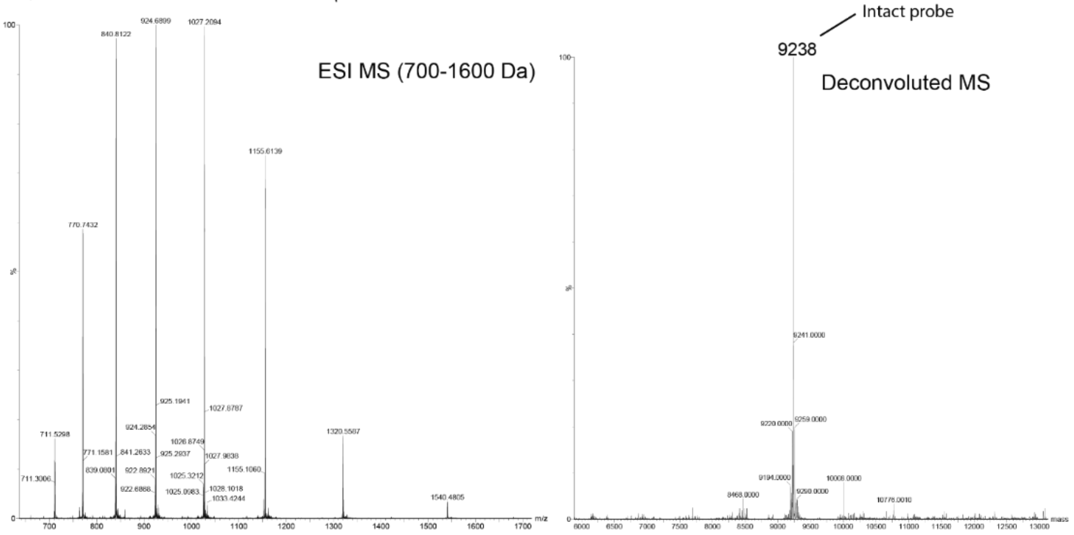

A) 15 min 300 µM GSH

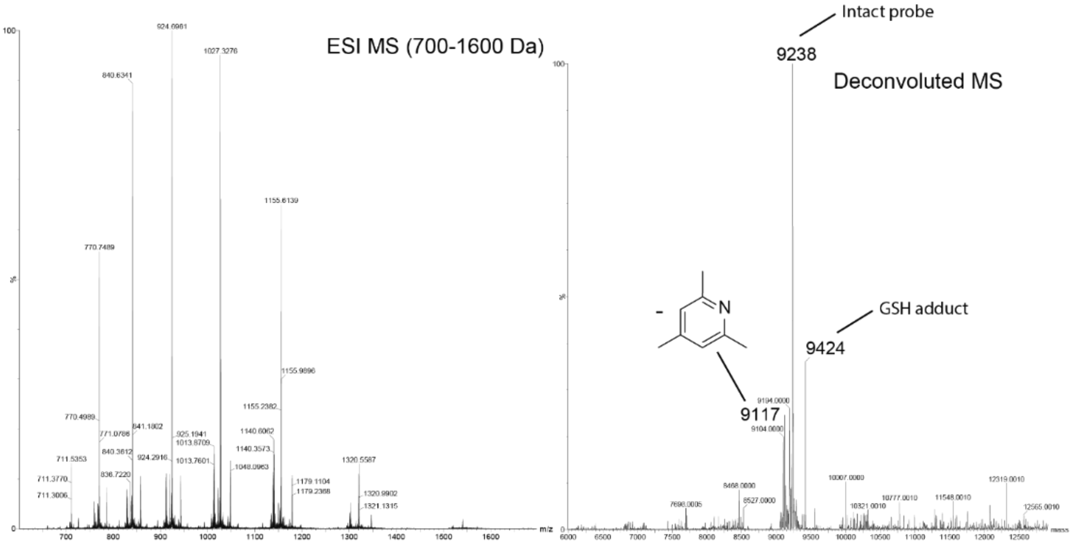

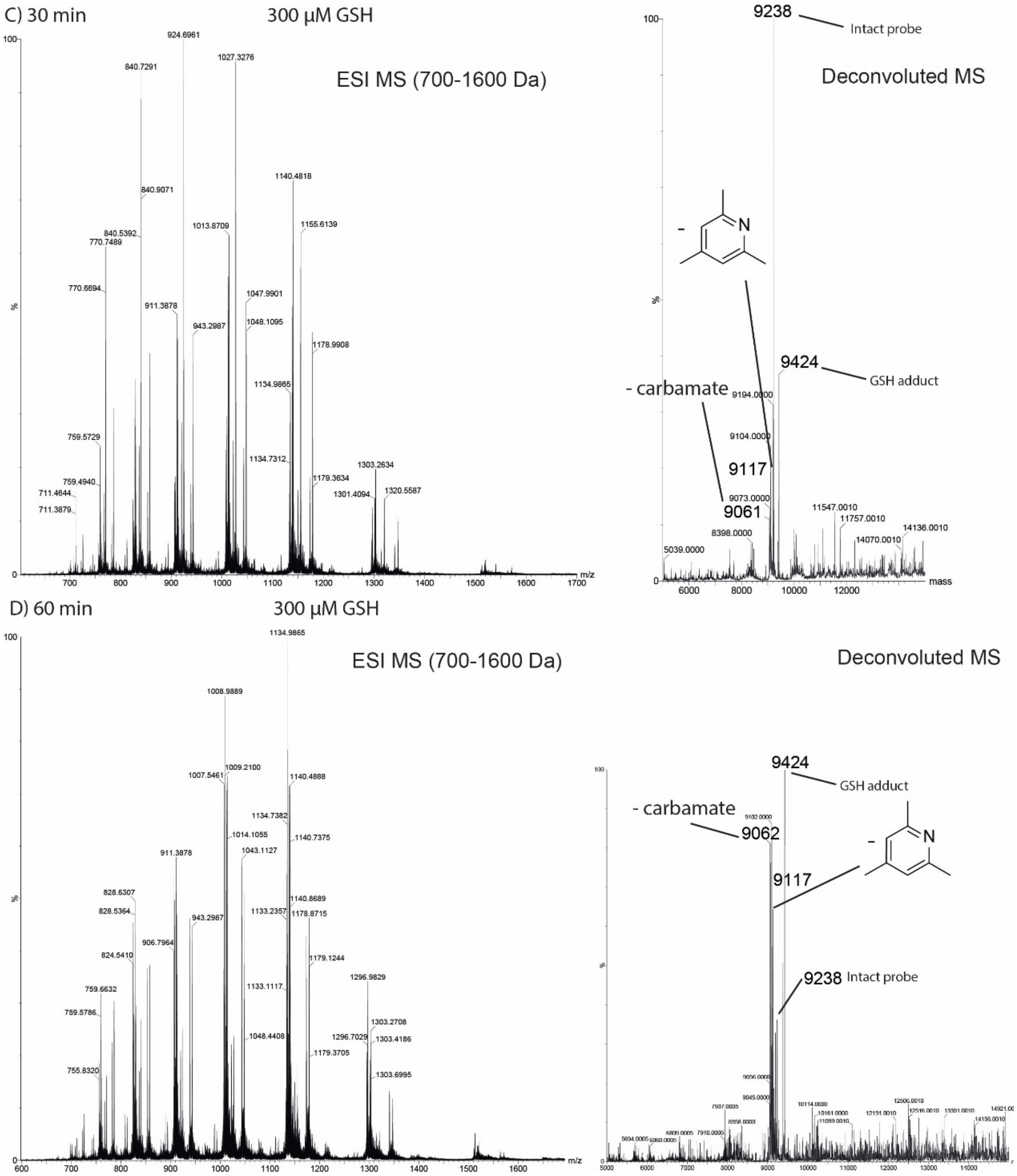

Supplementary Fig. 2. HRMS analysis of the activation of probe **5**. **A**) Probe **5** activation via 1 min of irradiation (302 nm) in the presence of GSH (300 μM) as additive. Predominantly intact probe (M + H^+) is detected, **B**) The photoactivation reaction analyzed after 15 min. Probe activation is observed via loss of the pyridinium moiety (M + H^+ = 9117, Δ = 120) as well as GSH addition (M + H^+ = 9423, Δ = 185, concomitant loss of pyridinium moiety) are observed, **C**) The photoactivation reaction analyzed after 30 min. Probe activation is observed via loss of the pyridinium moiety (M + H^+ = 9117, Δ = 120) and concomitant loss of the carbamate moiety (M + H^+ = 9062). Residual starting material (M + H^+ = 9236) is as well detected, **D**) The photoactivation reaction analyzed after 60 min.

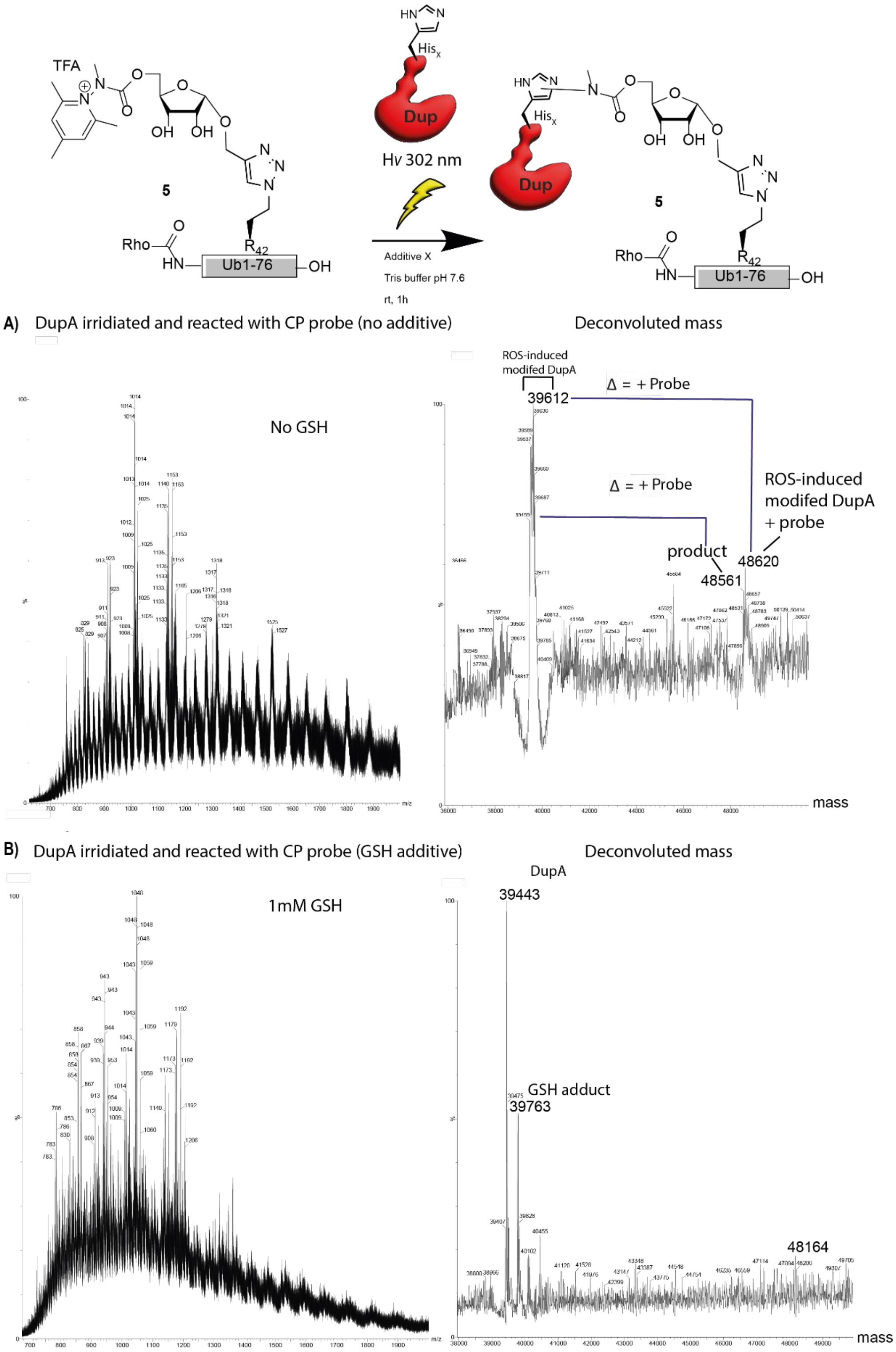

Supplementary Fig. 3. HRMS of the reaction between probe **5** and DupA irradiated for 1 hour at 302 nm. The reaction is monitored in the **A**) absence and, **B**) presence of GSH and analyzed by HRMS.

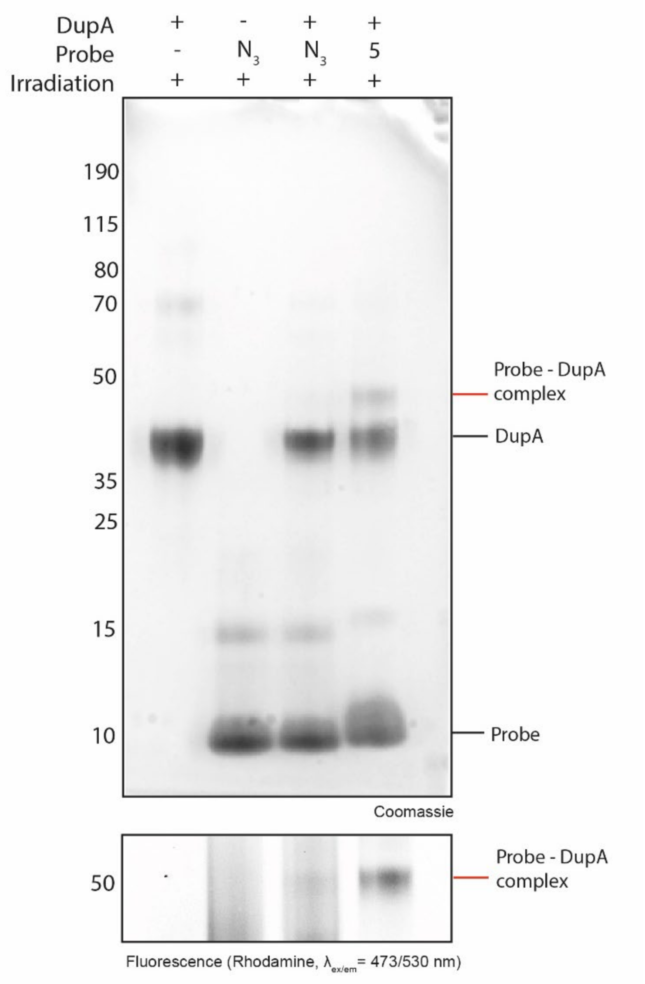

Supplementary Fig. 4. DupA does not crosslink to Rhodamine-(Arg42 to azido homoalanine) Ub_{76} (annotated as N_3) upon photoactivation, whereas it does with probe **5**. Irradiation is performed for 1 hour at 302 nm. Upper panel: Coomassie stained, bottom panel: Rhodamine fluorescence scan ($\lambda_{ex/em}$ = 473/530 nm).

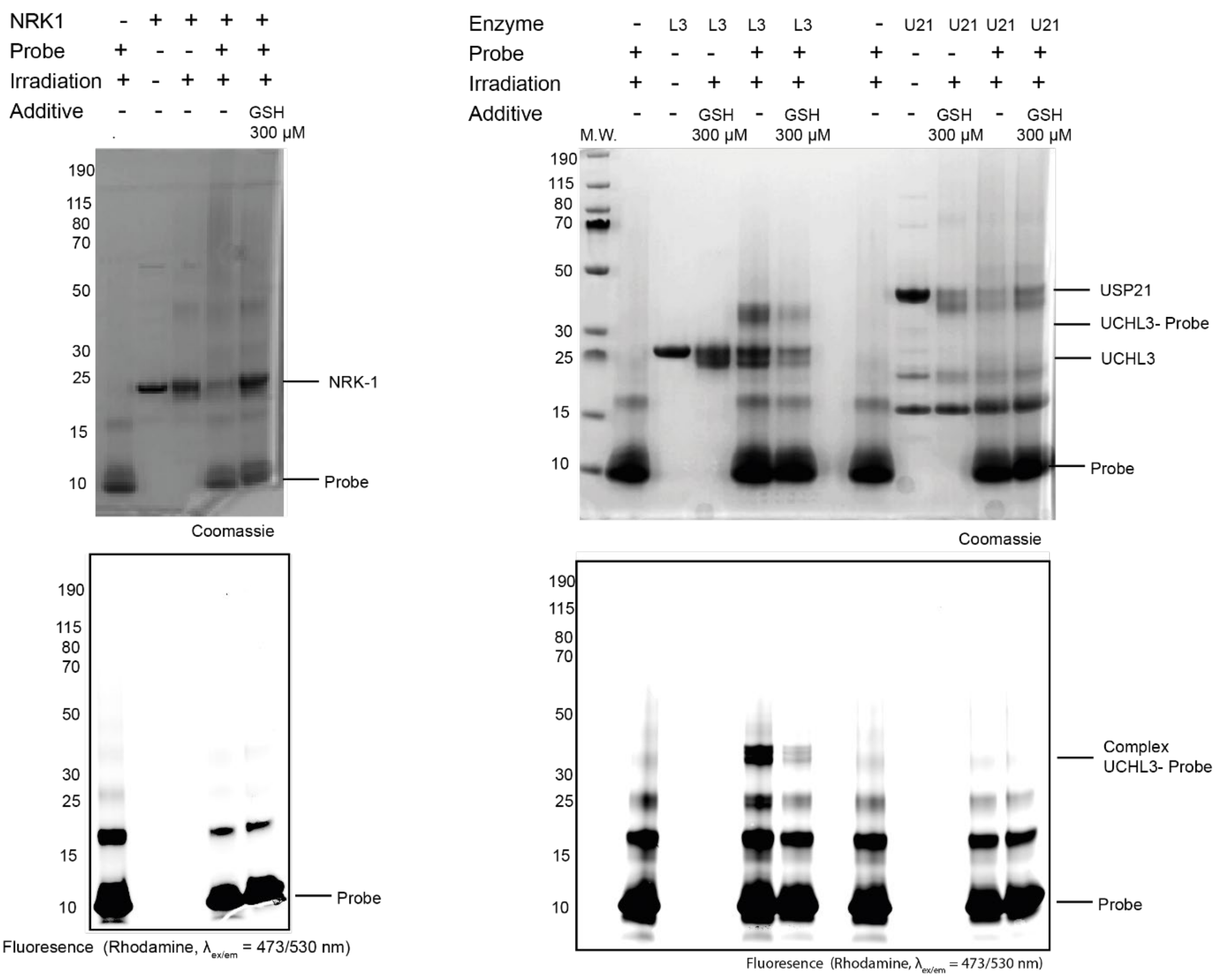

Supplementary Fig. 5. Ub pyridinium probe **5** binds UCH-L3 upon photoactivation. SDS-PAGE showing complex formation between probes **5** and UCH-L3 whereas no reaction for NRK-1 and USP21 is observed. Irradiation is performed for 1 hour at 302 nm. Upper panel: Coomassie stained, bottom panel: Rhodamine fluorescence scan ($\lambda_{ex/em}$ = 473/530 nm).

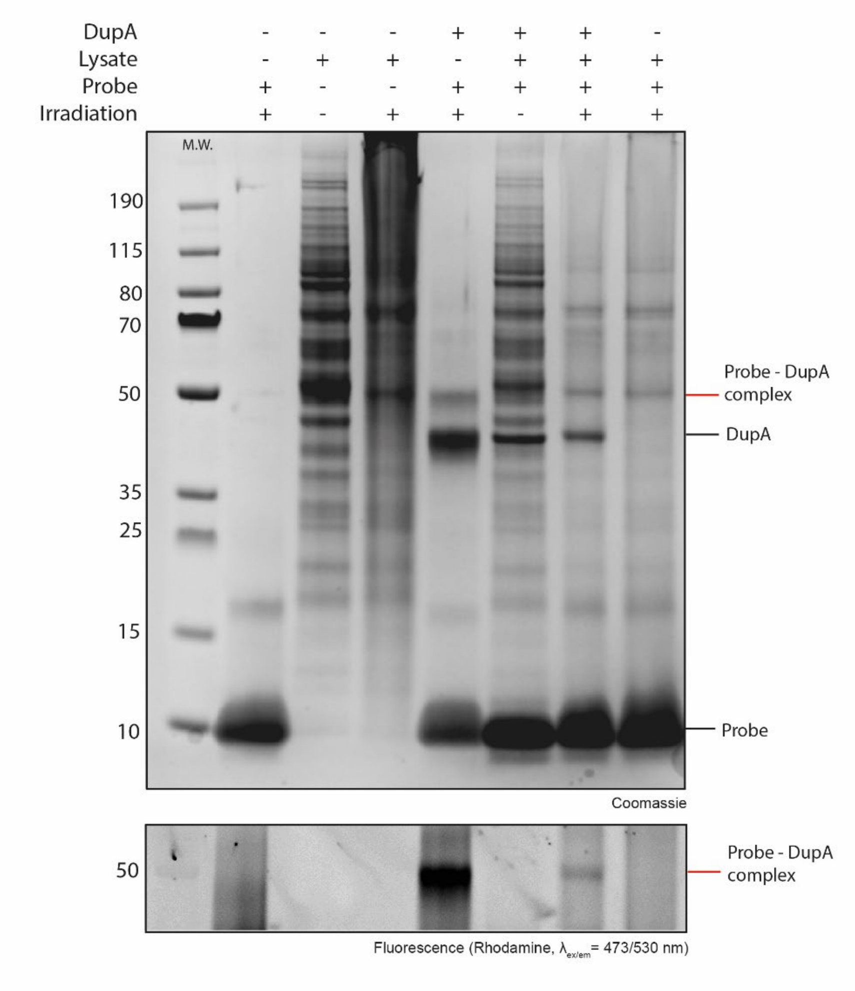

Supplementary Fig. 6. DupA (2 μM spiked in) labelling in HEK293T cell lysate using Probe **5** (200 μM). Irradiation is performed for 15 min at 302 nm. Upper panel: Coomassie stained, bottom panel: Rhodamine fluorescence scan ($\lambda_{ex/em}$ = 473/530 nm).

Photochemical cross-coupling of pyridinium probe 5 to Legionella effector enzymes, NRK-1 and DUBs

To a 96-wells plate containing buffer (20 mM TRIS, 150 mM NaCl, pH 7.6) the enzyme (Rho-Ub Arg42 to azido homoalanine, DupA, SdeA, NRK-1, UCHL-3 or USP-21, 10 μM), probe **5** (100 μM, 10 eq.) and optionally an additive (GSH, 1 mM or 300 μM) were added. The reactions were performed in a total volume of 50 μL and preincubated on ice for 15 min before irradiation using a 302 nm UV lamp (Analytikjena, UVP 3UV Lamp, 8 W), positioned on top of the 96-wells plate for 15 min, 30 min or 1 hour. The reaction mixture was then analyzed via LC/MS or SDS-PAGE. All experiments were performed with N=3 using the same protocol, and a single experiment is depicted as representative figure.

Photochemical cross-coupling of pyridinium probe 5 to DupA spiked into HEK293T lysate

To a 96-wells plate containing HEK293T cell lysate the enzyme (DupA, 2 μM), probe **5** (200 μM, 100 eq.) and GSH (300 μM) were added. The reactions were performed in a total volume of 25 μL and preincubated on ice for 15 min before irradiation using a 302 nm UV lamp (Analytikjena, UVP 3UV Lamp, 8 W), positioned on top of the 96-wells plate for 15 min. The reaction mixture was then analyzed directly via SDS-PAGE.

General synthetic procedures

All reagents were used as received unless stated otherwise. Solvents used in synthesis were dried and stored over 4Å molecular sieves, except for MeOH and MeCN which were stored over 3Å molecular sieves. Triethylamine (TEA) and diisopropylethylamine (DIPEA) were stored over KOH pellets. TLC analysis was performed on Macherey-Nagel aluminium sheets (silica gel 60 F_{254}). TLC was used to visualize compounds by UV at wavelength 254 nm and by spraying with either cerium molybdate spray (25 g/L $(NH_4)_6Mo_7O_{24}$, 10 g/L $(NH_4)_4Ce(SO_4)_4 \cdot H_2O$ in 10% H_2SO_4 water solution) or $KMnO_4$ spray (20 g/L $KMnO_4$ and 10 g/L K_2CO_3 in water) followed by charring at c.a. 250 °C. NMR spectra were recorded on a Bruker AV-300 NMR. Chemical shifts (δ) are given in ppm relative to tetramethyl silane. Coupling constants (*J*) are given in Hz. All given ^{13}C-APT spectra are proton decoupled.

LC-MS measurements and HPLC purifications

LC-MS measurements were conducted on a Waters ACQUITY UPLC H-class System equipped with a Waters ACQUITY Quaternary Solvent Manager (QSM), Waters ACQUITY UPLC Photodiode Array (PDA) eλ Detector (λ = 210-800 nm), Waters ACQUITY UPLC Protein BEH C18 column (1.7 μM, 2.1 x 50 mm) and LCT Premier Orthogonal acceleration Time of Flight Mass Spectrometer (m/z = 100-1600) in ES+ mode. Samples were run for 3min at 40 °C using 2 mobile phases: A: MQ + 0.1% formic acid, B: MeCN + 0.1% formic acid. Gradient: 0 - 95% B at a flow rate of 0.5 mL/min. Data processing was performed using Waters MassLynx Mass Spectrometry Software 4.1 (deconvolution with MaxEnt1 function).

HPLC purification was performed on a **A)** Shimadzu semi-preparative RP-HPLC system, equipped with a Waters C18-Xbridge 5 μm OBD (10 x 150 mm) column at a flowrate of 6.5 mL/min. using 2 mobile phases: A: MQ + 0.05% FA, B: MeCN + 0.05 % FA. Gradient: 10 -> 70% B. HPLC system **B**) Waters preparative RP-HPLC system, equipped with a Waters C18-Xbridge 5 μm OBD (30 x 150 mm) column at a flowrate of 37.5 mL/min using 3 mobile phases: A: MQ, B: CH_3CN and C: 1% TFA in MQ. Gradient: 20 -> 45% B, 5% C. High resolution mass spectra were recorded on a Waters XEVO-G2 XS Q-TOF mass spectrometer equipped with an electrospray ion source in positive mode (source voltage 3.0 kV, desolvation gas flow 900 L/hr, temperature 250 °C) with resolution R = 22000 (mass range m/z = 50-2000) and 200 pg/μL Leu-Enk (m/z = 556.2771) as a "lock mass".

Chemical synthesis

α-1'-O-propargyl-5'-O-(2,4,6-trimethyl-1-pyridin-1-ium)-ribofuranoside (4)

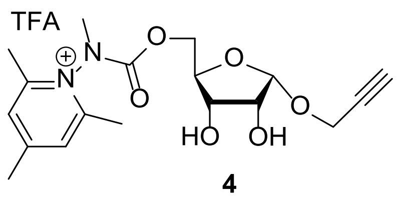

4

Pyridinium **4** was synthesized by adapting the procedure from Tower *et al*[37]., as follows:

α-1'-O-propargyl-2',3'-*O-iso*propylidene-ribofuranose **1** (20 mg, 0.087 mmol) and 1,1-carbonyldiimidizole (28.4 mg, 0.18 mmol, 2 eq.) were added to a flask and co-evaporated with

toluene. Subsequently, the reactants were placed under argon atmosphere, dissolved in anhydrous MeCN (0.44 mL) and the reaction stirred for 4.5 h. Next, additional MeCN (0.2 mL) was added followed by methyl hydrazine (9.2 µL, 0.18 mmol, 2 eq.), and the reaction mixture was vigorously stirred at rt. After 1h, complete consumption of the starting material was detected by LC-MS. The reaction mixture was then concentrated *in vacuo* and co-evaporated with toluene to a afford the crude hydrazine **2**. The crude hydrazine and 2,4,6-trimethylpyrylium tetrafluoroborate (18.3 mg, 0.087 mmol, 1 eq.) were subsequently co-evaporated with toluene, placed under argon atmosphere and dissolved in EtOH (0.15 mL) . After stirring the reaction mixture overnight, the red reaction mixture was concentrated *in vacuo*. Precipitation was attempted in DCM:EtOH (2:1 v/v ratio), but proved challenging at the performed reaction scale. The crude pyridinium **3** was then dissolved in a mixture of AcOH:H_2O (1 mL, 1:1 v/v) and stirred at 60 °C. After stirring for 2 days, LC-MS indicated complete deprotection of the i*so*propylidene group (M + H^+ = 365). The reaction mixture was diluted with toluene and concentration *in vacuo*. Subsequent purification of the crude by preparative HPLC afforded pyridinium **4** (5.1 mg, 0.014 mmol, 16%) as a pale orange solid. **4** exists as a 1:1 mixture of rotamers at room temperature. ^{19}F NMR indicates **4** to be a TFA salt. ^{1}H NMR (300 MHz, MeOD) δ 7.90 (d, *J* = 8.7 Hz, 1H), 5.30 (d, *J* = 4.3 Hz, 0.5H), 5.09 (d, *J* = 4.2 Hz, 0.5H), 4.62 – 4.47 (m, 1H), 4.45 – 4.37 (m, 2H), 4.37 – 4.34 (m, 1H), 4.34 – 4.25 (m, 2H), 4.22 – 4.15 (m, 1H), 4.14 – 4.00 (m, 2H), 2.95 (td, *J* = 2.4, 0.8 Hz, 1H), 2.79 (dt, *J* = 7.5, 0.5 Hz, 3H), 2.76 (dd, *J* = 4.9, 0.6 Hz, 3H), 2.69 (dt, *J* = 4.5, 0.6 Hz, 3H). ^{13}C NMR (75 MHz, Methanol-d_4) δ 164.3, 159.0, 130.2, 101.9, 83.2, 82.8, 76.6, 73.1, 71.5, 69.2, 55.7, 38.3, 22.4, 19.2. ^{19}F NMR (282 MHz, MeOD) δ -76.98.

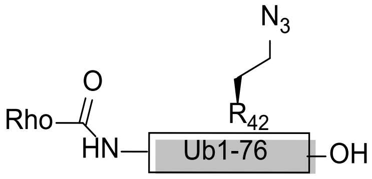

Rhodamine-(Arg42 → azido homoalanine mutation) **Ub$_{76}$**

Is synthesized according to a reported procedure: Kloet, M.S. *et al.* (2024) Development of covalent probes to capture Legionella pneumophila effector enzymes, *Biorxiv* [Preprint]. doi:10.1101/2024.03.19.585531.

Rhodamine-(Arg42 → triazole linked 5'-O-pyridinium riboside) Ub_{76} (5)

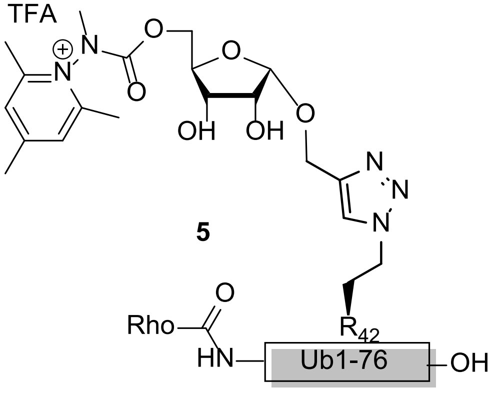

5

Pyridinium riboside 4 (15 µL, 0.15M in DMSO, 2.25 µmol, 4.0 eq) was added to a prepared solution of rhodamine ubiquitin bearing a Arg42 to azido homoalanine mutant ($M + H^+$ = 8873, 5 mg, 0.56 µmol, 1 eq.) in DMSO (45 µL) and TRIS buffer (1.5 mL, 20 mM TRIS/150 mM NaCl, pH 7.6). To this was added 15 µL of freshly prepared click-mixture (1:1:1 v/v/v, $CuSO_4$ (100 mM in H_2O): Sodium Ascorbate (600 mM in H_2O): TBTA ligand (100 mM in MeCN). The reaction mixture was shaken at 37 °C for 30 min before the addition of another portion of freshly prepared click mixture (30 µL). After shaking for another 30 min, LC-MS verified complete conversion to the product (mass found: ($M + H^+$ = 9237). The conjugate was purified by RP-HPLC. Pure fractions were pooled and lyophilized obtaining ubiquitin-ribose conjugate **5** (3.1 mg, 0.033 µmol, 60%) as a red powder. HRMS: $[C_{414}H_{658}N_{111}O_{128}^+ + 7H]^{7+}$ found: 1320.6249, calculated: 1320.7814. $[C_{414}H_{658}N_{111}O_{128}^+ + 8H]^{8+}$ found: 11455.6383, calculated: 1155.8088. $[C_{414}H_{658}N_{111}O_{128}^+ + 9H]^{9+}$ found: 1027.2579, calculated: 1027.4967. $[C_{414}H_{658}N_{111}O_{128}^+ + 10H]^{10+}$ found: 924.5728, calculated: 924.8470. $[C_{414}H_{658}N_{111}O_{128}^+ + 11H]^{11+}$ found: 840.6064 calculated: 840.8609.

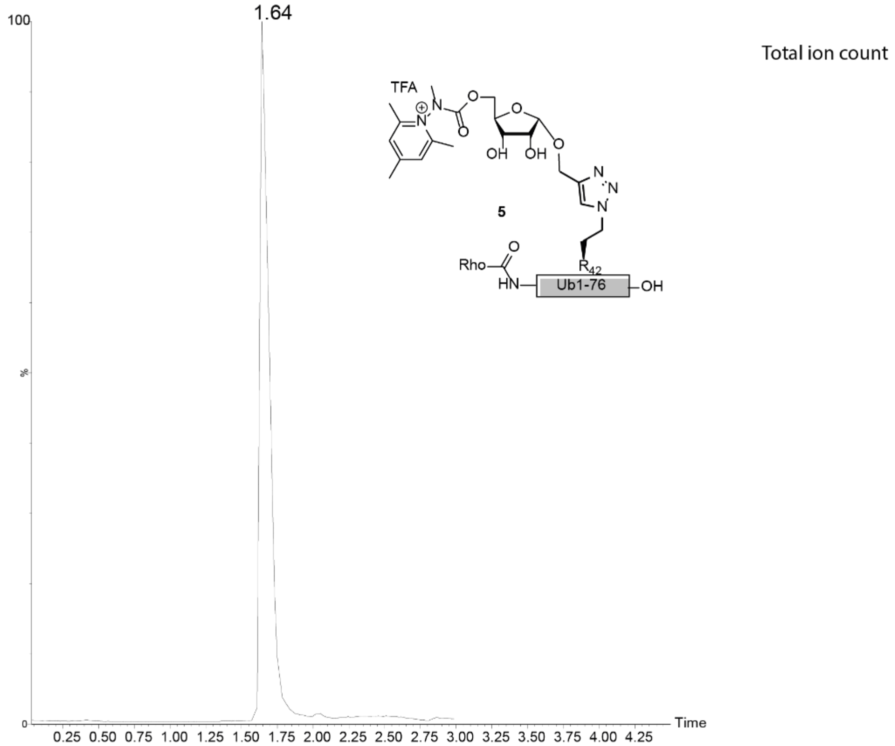

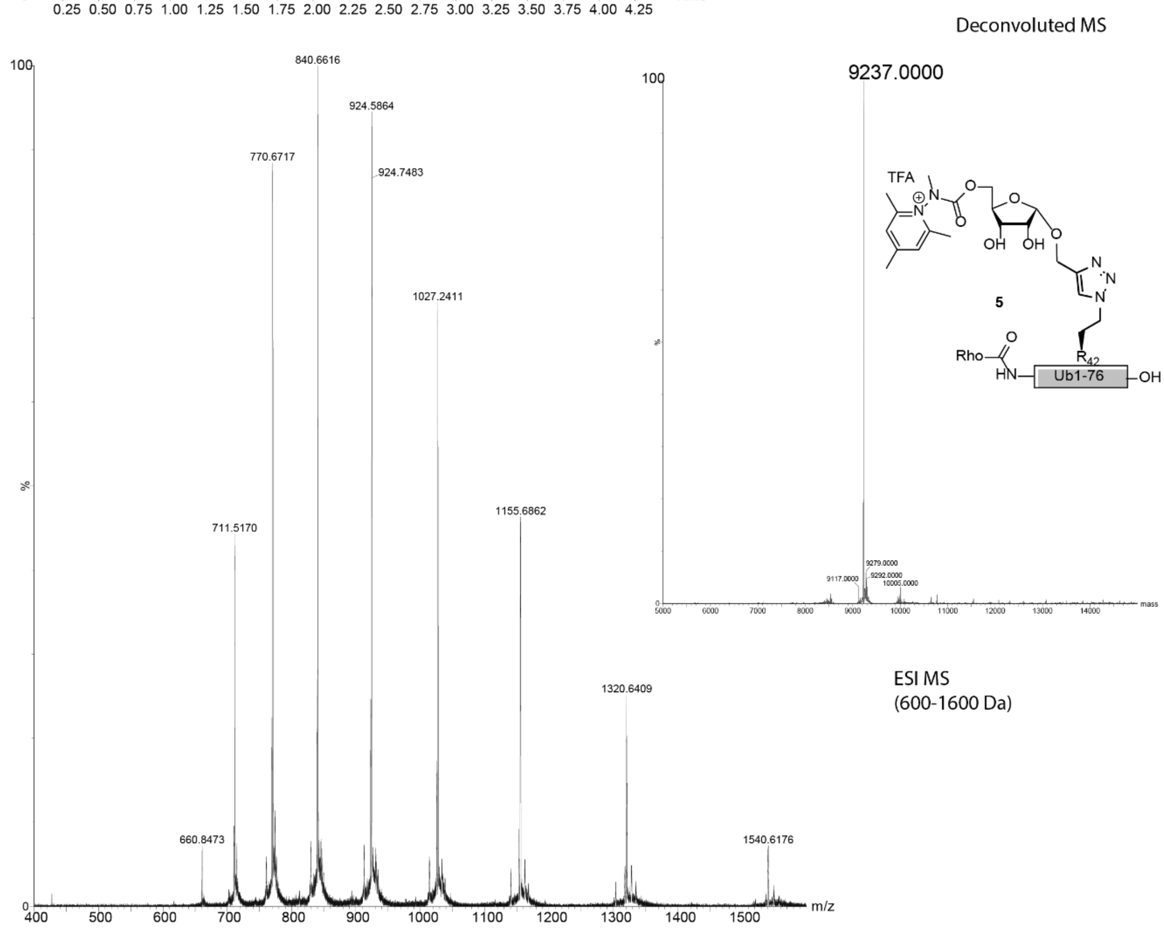

HRMS analysis of Probe **5**.
